# The Comparison of Gut Bacteria Communities and the Functions Among the Sympatric Grasshopper Species From the Loess Plateau

**DOI:** 10.3389/fmicb.2022.806927

**Published:** 2022-04-05

**Authors:** Lu Zhao, Wen-Qiang Wang, Sheng-Quan Xu, De-Long Guan

**Affiliations:** ^1^College of Life Science, Shaanxi Normal University, Xi’an, China; ^2^College of Life Sciences, Yan’an University, Yan’an, China

**Keywords:** gut bacteria, grasshopper, comparison, ecological environment, Loess Plateau

## Abstract

Gut bacteria exert effects on the health and fitness of their insect hosts. Grasshoppers are an important part of the grassland ecosystem and provide important ecosystem services. As the most valuable feature in grassland ecosystem, the compositions and potential influences of gut bacterial in herbivorous grasshoppers in the same ecological environment are essential but undetermined. To facilitate such studies, we collected nine species of grasshoppers (*n* = 110) from a rebuild grassland on the Loess Plateau in northern Shaanxi, China, which is a representative area of ecosystem restoration model. We characterized the composition and function of the gut bacteria. We found that 326 OTUs were exhibited in all grasshoppers in which *Enterobacter*, *Pantoea*, *Bacillus*, and *Spiroplsma* are dominant. Among them, 18 OTUs were shared across all nine species of grasshoppers. The predicted function showed that the majority function of those OTUs were involved in survival dependent processes including membrane transport, carbohydrate metabolism, amino acid metabolism, and DNA replication and repair. The composition of gut bacteria is specific to each grasshopper species, and the bacteria community is most various in *Trilophidia annulata*. These results highlight the gut bacterial community diversity in different grasshopper species. Our findings are necessary for better understanding the relationships between this important herbivorous insect and their microbiomes and have the potential contribution of evaluating the revegetation and ecosystem management in this area.

## Introduction

Recently, the increasing interest in the research of insect microbiome has helped to reveal new insights into insect ecology (Muratore et al., 2020a). As an important part of the insect microbiome, gut bacteria played an essential role in the life course of their insect hosts, including nutrient absorption (Muratore et al., 2020b), behavioral changes (Dillon et al., 2000), improved reproduction (Kaltenpoth et al., 2009), and protection from environmental pathogens (Paniagua Voirol et al., 2018). In agricultural production, the interaction of gut bacteria with herbivores and pollinators can affect the yield of crops (Ray et al., 2007; Fierer et al., 2009). Gut bacterial communities and their insects are regarded as biogeochemical cycling mediators in the ecological environment (Ricci et al., 2012). Insect gut bacteria, for example, can regulate the decomposition of plant biomass, carbon cycling, and nitrogen fixation rates (Rajagopal, 2009).

Numerous studies of gut bacteria of insects have focused on several major insect orders, including Coleoptera (Scott et al., 2008; Kim et al., 2017), Orthoptera (Rogers et al., 2003; Dillon et al., 2005), Hymenoptera (Russell et al., 2009; Koch et al., 2013; Saraiva et al., 2015), Diptera (Behar et al., 2005; Dong et al., 2009; Wang et al., 2013), Lepidoptera (Ruokolainen et al., 2016), and Blattaria (Hongoh, 2010; Brune, 2014). Most of these studies mainly focused on the functional analysis of single species of gut bacteria of insects or compared the community structure and function of gut bacteria among different insect species. For instance, Shi et al. (2013) compared the composition and functionality of the gut bacteria in grasshopper, cutworm, and termite. Warnecke et al. (2007) had found that the gut bacteria of termite could assist the host in degrading lignocellulose, so as to make up for the deficiency of the termite. Engel and Moran (2013) found that the *Gilliamella* of the bees’ gut bacteria were able to encode pectin degrading enzymes that help the bees in breaking down the pollen wall. Sharon et al. (2010) found a close relationship between sexual selection and gut bacteria of *Drosophila*.

Grasshoppers belong to Orthoptera and are the main herbivores in the global grassland ecosystem (Muratore et al., 2020a). Due to their consumption of large amounts of plant biomass, grasshoppers are the most important primary consumers and play an important role in maintaining ecological balance (Aiello, 2005; Sun et al., 2015). Grasshoppers are also considered a key indicator of the stability of grassland ecosystems, especially in threatened habitats such as grasslands (Báldi and Kisbenedek, 1997; Muratore et al., 2020a). However, the exploration of the resident gut bacterial community of grasshopper is still limited. This study provided the characterization of the gut bacterial communities of grasshopper with similar feeding habits in the same specific ecological environment, which could help us to better understand the role that gut bacteria played on insect hosts.

In order to provide a broader environmental background, we chose the different species of herbivorous grasshoppers living in the Loess Plateau. The Loess Plateau (35.3′–39.17′N, 107.20′–111.04′E) of North Shaanxi Province in China is an area of approximately 92,521.4 km^2^, which is one of the most serious areas of ecological deterioration and soil erosion in the world (Fu et al., 2000). This region has a semi-arid continental climate. About 3 or 4 decades ago, due to deforestation, the forest had virtually disappeared. Therefore, the Chinese government implemented the policy of grain for green project in the 1990s to increase the rate of vegetation coverage (Shao et al., 2021). Until now, the existing plants are mainly composed of grasslands and shrubs (Qiu et al., 2001). Common plants in this area, including *Stipa capillata*, *Artemisia giraldii*, *Bothriochloa ischaemum*, and *Artemisia gmelinii*, provide a suitable living environment for grasshoppers (Fu et al., 2004; Gong et al., 2006). Common grasshoppers in this area include the genera of *Oedaleus*, *Calliptamus*, *Atractomorpha*, *Epacromius, Trilophidia*, and *Oxya* (Otte, 2005). These grasshoppers primarily feed on plants in the families of Gramineae and Asteraceae.

Nine species of grasshoppers in North Loess Plateau were collected in this study, including *Heteropternis robusta* Bey-Bienko, 1951; *Acrida oxycephala* Pallas, 1771; *Epacromius coerulipes* Ivanov, 1888; *Eremippus mongolicus* Ramme, 1952; *Trilophidia annulata* Thunberg, 1815; *Oedaleus infernalis* Saussure, 1884; *Atractomorpha sinensis* Bolívar, 1905; *Aiolopus tamulus* Fabricius, 1798; and *Calliptamus abbreviatus* Ikonnikov, 1913. We sequenced the 16S rRNA V3–V4 region of gut bacteria from nine grasshopper species to investigate (1) the composition of bacterial communities in different grasshoppers; (2) these gut bacteria function; (3) the comparison of gut bacteria communities across nine grasshopper species in the same area.

## Materials and Methods

### Sample Collection

A total of 110 adult (the fifth and sixth instar) individuals of grasshoppers were collected from Yuyang County and Yulin City of Shaanxi Province (Longitude: 109.73′ E, latitude: 38.35′ N). They are from different species: *Heteropternis robusta* (10 individuals), *Acrida oxycephala* (10 individuals), *Epacromius coerulipes* (10 individuals), *Eremippus mongolicus* (10 individuals), *Trilophidia annulata* (10 individuals), *Oedaleus infernalis* (30 individuals), *Atractomorpha sinensis* (10 individuals), and *Calliptamus abbreviatus* (10 individuals).

### Isolation of the Guts From the Grasshoppers

We divided all the grasshoppers into three groups (Group A, Group B, and Group C). Group A includes five species belonging to different subfamilies, which are *A. sinensis* (Pyrgomorphinae), *C. abbreviatus* (Calliptaminae), *E. mongolicus* (Gomphocerinae), *A. oxycephala* (Acridinae), and *O. infernalis* (Oedipodinae). The five species of group B are collected from the *E. coerulipes*, *A. tamulus*, *H. robusta*, *O. infernalis*, and *T. annulate*, and they belong to Oedipodinae. Group C includes three samples collected from the fore-gut and mid-gut of *O. infernalis* (Supplementary Table 1). Dissection was performed on each grasshopper by removing the entire gut before extracting their bacteria. All samples were firstly quick-frozen in liquid nitrogen for 2–3 min, and then they were disinfected with 70% alcohol for 1 min. The gut of grasshoppers was extracted by sterile scissors. All the anatomical procedures were performed on clean bench library preparation.

### DNA Extraction and PCR Amplification of 16S rRNA

DNA was extracted from grasshoppers using the E.Z.N.A.^®^ Soil DNA Kit (Omega Bio-Tek, Norcross, GA, United States) according to the manufactures’ manual. The V3–V4 region of the bacteria 16S ribosomal RNA gene was amplified by PCR using primers 341F (5′-barcode- CCTACGGGAGGCAGCAG-3′) and 806R (5′-GGACTACHVGGGTWTCTAAT-3′), where barcode is an eight-base sequence unique to each sample. PCR reactions were performed in a triplicate 20-μl mixture containing 4 μl of 5 × FastPfu Buffer, 2 μl of 2.5 mM dNTPs, 0.8 μl of each primer (5 μM), 0.4 μl of FastPfu Polymerase, and 10 ng of template DNA at 95°C for 2 min, followed by 25 cycles at 95°C for 30 s, 55°C for 30 s, 72°C for 30 s and a final extension at 72°C for 5 min. The PCR amplicon products were extracted from 2% agarose gels and purified using the AxyPrep DNA Gel Extraction Kit (Axygen Biosciences, Union City, CA, United States) according to the manufacturer’s instructions and quantified using QuantiFluor™ -ST (Promega, Madison, WI, United States).

### 16S rRNA Sequencing

Purified PCR products were quantified by Qubit^®^3.0 (Life Invitrogen) and every 24 amplicons whose barcodes were different were mixed equally. The mixed DNA product was used to construct Illumina Pair-End library following Illumina’s genomic DNA library preparation procedure. Then, the amplicon library was paired-end sequenced (2 × 250 bp) on an Illumina HiSeq 2000 platform (McHardy et al., 2013) according to the standard protocols.

Overall, for each species, over 30 Mb raw data were retrieved and saved in two separated files which contain the forward and reversed reads. These raw data were initially conducted by the Trimmomatic software (Bolger et al., 2014) to perform the quality check. In this step, low-quality reads including the adaptor contaminated reads, along with the reads that have quality score Q20 less than 20, or length less than 50 bp, were all removed and those retained were restored as clean reads.

## Data Analysis

To perform the statistical analysis of these clean reads, we used the QIIME software package environment (version 2.01) (Bolyen et al., 2019). First of all, the forward and reverse reads were imported and merged using the Join_paired_end.py script, then the reads were demultiplexed by different types of barcodes using the split_libraries_fastq.py script. The generated demultiplexed reads were saved in one single fasta format file named seq.fna. This file was compared with the downloaded core_set_aligned.fasta.inputed file^1^ to identify and remove the chimeric reads. After these steps, we obtained the organized sequences, which are suitable for OTU classification. We use the UPARSE pipeline (version 7.1)^2^ (Edgar, 2013) to classify all these sequences into a OTU table with the threshold of 97%. The OTUs were picked by both the opened and the closed methods using the SILVA (SSU126) 16S rRNA database and Greengenes 13.8 (DeSantis et al., 2006) as reference. The open-picked and closed-picked OTUs were used for different analysis purposes. The open-picked OTUs comprise new bacteria information that lack annotation, and they are more sufficient in generating more accurate OTUs which are better for further alpha and beta analysis and phylogenetic affiliation of each 16S rRNA genes. The RDP Classifier^3^ software (Cole et al., 2005) is used to conduct this analysis and the confidence threshold is 70%. The closed-picked OTUs only contain those with KEGG annotation, which were later used in PICRUSt analysis.

### Functional Predictions

PICRUSt (Phylogenetic Investigation of Communities by Reconstruction of Unobserved States) (Douglas et al., 2018) was used to predict the function of bacterial communities based on 16S rRNA sequencing data. For this analysis, the OTUs were obtained from the Greengenes 13.8 (DeSantis et al., 2006) reference database. The mapped OTUs were further characterized and annotated using the KEGG database.

## Results

### Sequencing and Diversity of Gut Bacteria Community

On sequencing of the 16S rRNA V3–V4 region of nine species of grasshoppers, respectively, a total of 461,372 high-quality sequences were obtained after basic processes and a series of quality filtering. The average length of reads was 427 bp. These high-quality sequences which were clustered into OTUs were at 97% similarity threshold. The taxonomic classification of OTUs was performed using RDP Classifier (see text footnote 3). A total of 326 OTUs were identified from all gut bacteria of grasshoppers (Supplementary Tables 2, 3).

Rarefaction curve can be used to determine whether the current sequencing depth of each sample is sufficient to reflect the microbial diversity contained in the community sample. In this study, the Shannon index rarefaction curves in all individuals reached the plateau (Supplementary Figure 1), indicating that most of the bacterial diversity in all grasshoppers was captured in the current sequencing. Other undiscovered rare species will not affect our conclusion based on diversity-index.

### The Composition of the Gut Bacteria Community

**FIGURE 1:**
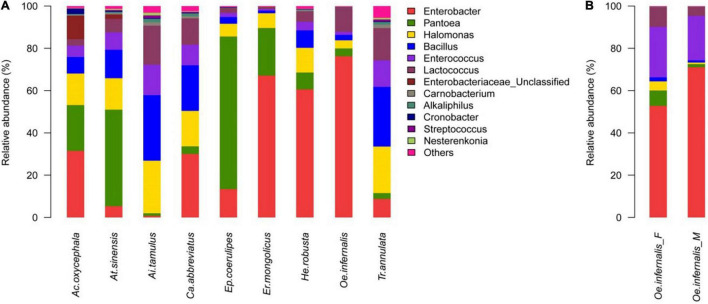
Genus-Level Gut Bacteria Composition of Grasshoppers. **(A)** Relative contribution of the gut bacteria in *Ac. oxycephala*, *At. sinensis*, *Ai. tamulus*, *Ca. abbreviatus, Ep. coerulipes*, *Er. mongolicus*, *He. robusta*, *Oe. infernalis*, and *Tr. annulata*. **(B)** Relative contribution of the gut microbiota in fore-gut and mid-gut of *Oe. infernalis*.

According to the results of species annotation and statistical analysis, nearly all reads were assignable to 20 phyla, 210 genera. The distribution of dominant phyla and genera in nine species of grasshoppers was as follows: except for *A. tamulus* and *T. annulata*, the main phyla of the other seven species of grasshoppers were Proteobacteria. At the genus level, the most dominant genus in *A. oxycephala*, *C. abbreviate*, *E. mongolicus*, *H. robusta*, and *O. infernalis* was *Enterobacter*, and the proportion was 31.50, 30.01, 64.16, 60.58, and 76.25%, respectively. The dominant genus in *A. sinensis* and *E. coerulipes* was *Pantoea*; the proportion was 45.64 and 72.18%, respectively. The dominant genus of *T. annulata* and *A. tamulus* was *Bacillus*; the proportion was 28.22 and 30.97%, respectively. More than 1% of the other genera were *Halomonas*, *Enterococcus*, and *Lactococcus* (Figure 1A).

Moreover, we found that the distribution of gut bacteria in for-gut and mid-gut of *O. infernalis* were different. The dominant genus was *Enterobacter*: the proportion was 52.78 and 71.01%, respectively (Figure 1B). This could be related to the function of different intestinal segments in host insects. The for-gut absorbs and stores food, while the mid-gut secretes digestive enzymes to digest food and absorb nutrients. The gut bacteria of insects interact with its host to perform these functions.

### The Comparative Analysis of Gut Bacteria Communities Among the Nine Grasshopper Species

**FIGURE 2:**
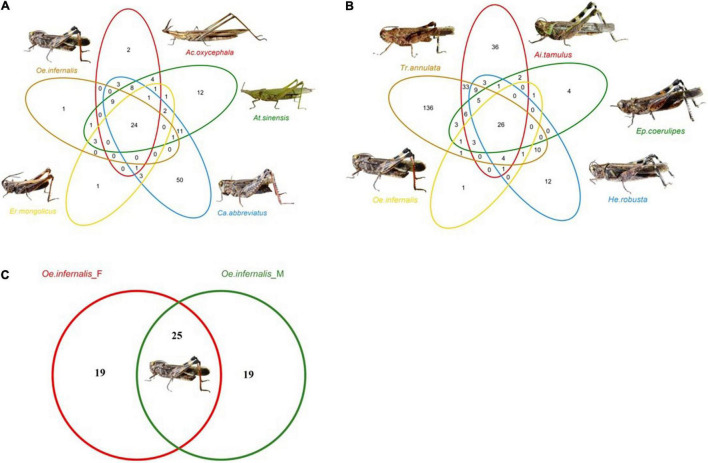
Venn diagrams show the common and unique OTUs of three groups. **(A)** The gut bacteria of five families, namely *At. sinensis* (Pyrgomorphinae), *Ca. abbreviatus* (Calliptaminae), *Er. mongolicus* (Gomphocerinae), *Ac. oxycephala* (Acridinae) and *Oe. infernalis* (Oedipodinae). **(B)** The gut bacteria of *Ep. coerulipes*, *Ai. tamulus*, *He. robusta*, *Oe. infernalis*, and *Tr. annulata* and they belonged to Oedipodinae. **(C)** The gut bacteria of fore-gut and mid-gut of *Oe. infernalis*.

We divided all the grasshoppers into three groups: Group A, Group B, and Group C (see Methods section “Isolation of the Guts From the Grasshoppers”). Then, we described the common and unique gut bacteria of nine grasshopper species in groups A, B, and C. A total of 140 OTUs were identified from group A, of which 24 OTUs were shared by them and the highest percentage of shared OTUs belonged to *Bacillus*. The number of unique OTUs in *C. abbreviatus*, *A. sinensis*, *A. oxycephala*, *O. infernalis*, and *E. mongolicus* was 50, 12, 2, 1, and 1, respectively (Figure 2A). A total of 300 OTUs were identified from group B, of which 26 OTUs were shared by them and the highest percentage of shared OTUs belonged to *Bacillus* and *Enterobacter*. The 136, 36, 12, 4, and 1 OTUs were uniquely identified with *T. annulata*, *A. tamulus*, *H. robusta, E. coerulipes*, and *O. infernalis*. The number of unique OTUs of *T. annulata* was significantly higher than that of other grasshopper species (Figure 2B). A total of 64 OTUs were identified from group C, of which 26 OTUs were shared by them and the highest percentage of shared OTUs belonged to *Bacillus* and *Enterobacter*. The 19 and 1 OTUs were uniquely identified of fore-gut and mid-gut in *O. infernalis* (Figure 2C). A total of 18 OTUs were shared across all grasshopper species, and the highest percentage of shared OTUs was *Bacillus*, *Enterobacter* and *Enterococcus*, respectively (Supplementary Table 4).

**FIGURE 3:**
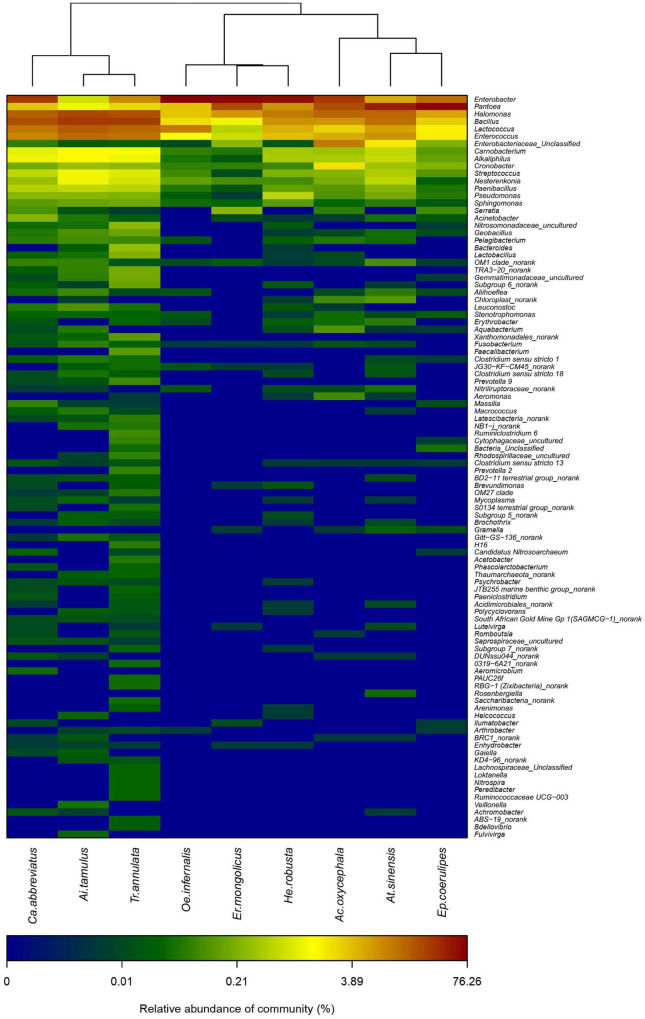
Heatmap showing the relative abundance of OTUs and clustering across nine species.

We calculated the Shannon, Simpson, and the Chao index of different grasshoppers to analyze the alpha diversity among the samples. The results showed that the alpha diversity of *T. annulata* was the highest, and the alpha diversity of *E. coerulipes* was the lowest (Table 1). Beta diversity of bacterial communities was assessed using non-metric multidimensional scaling (NMDS). NMDS ordination plots revealed clear differences in the community composition at the species level (Supplementary Figure 2). A heat map of representative genus indicated that all the grasshoppers were clustered into two categories according to the similarity of the gut bacteria composition of grasshoppers. The first category includes *C. abbreviatus*, *A. tamulus*, and *T. annulata.* The second category includes *O. infernalis*, *E. mongolicus*, *H. robusta*, *A. oxycephala*, *A. sinensis*, and *E. coerulipes.* The grasshoppers of the same family did not come together, indicating the differences between the gut bacterial communities of grasshoppers from the same family (Figure 3).

**TABLE 1:** Diversity Index of Gut Bacteria in Each Grasshopper.

Sample ID	α Diversity		
	Chao	Shannon	Simpson
*Ac. oxycephala*	69 (66, 83)	2.17 (2.16, 2.18)	0.1415 (0.1402, 0.1428)
*Ai. tamulus*	183 (172, 210)	2.06 (2.04, 2.07)	0.1835 (0.1818, 0.1851)
*At. sinensis*	93 (84, 121)	1.9 (1.88, 1.93)	0.2572 (0.2513, 0.2631)
*Ca. abbreviatus*	167 (153, 202)	2.24 (2.23, 2.25)	0.141 (0.1399, 0.1421)
*Ep. coerulipes*	84 (73, 115)	1.33 (1.32, 1.35)	0.4414 (0.4362, 0.4466)
*Er. mongolicus*	70 (58, 109)	1.38 (1.37, 1.4)	0.3602 (0.356, 0.3644)
*He. robusta*	153 (127, 212)	1.96 (1.94, 1.97)	0.2153 (0.2125, 0.2181)
*Oe. infernalis*	69 (57, 111)	1.44 (1.43, 1.45)	0.3771 (0.3723, 0.3819)
*Oe. infernalis_F*	97 (86, 129)	1.93 (1.92, 1.94)	0.1811 (0.1796, 0.1825)
*Oe. infernalis_M*	97 (83, 130)	1.45 (1.44, 1.46)	0.324 (0.321, 0.3269)
*Tr. annulata*	277 (270, 297)	2.36 (2.34, 2.38)	0.1648 (0.1629, 0.1667)

### Prediction and Differences in the Gut Bacteria Pathways

**FIGURE 4:**
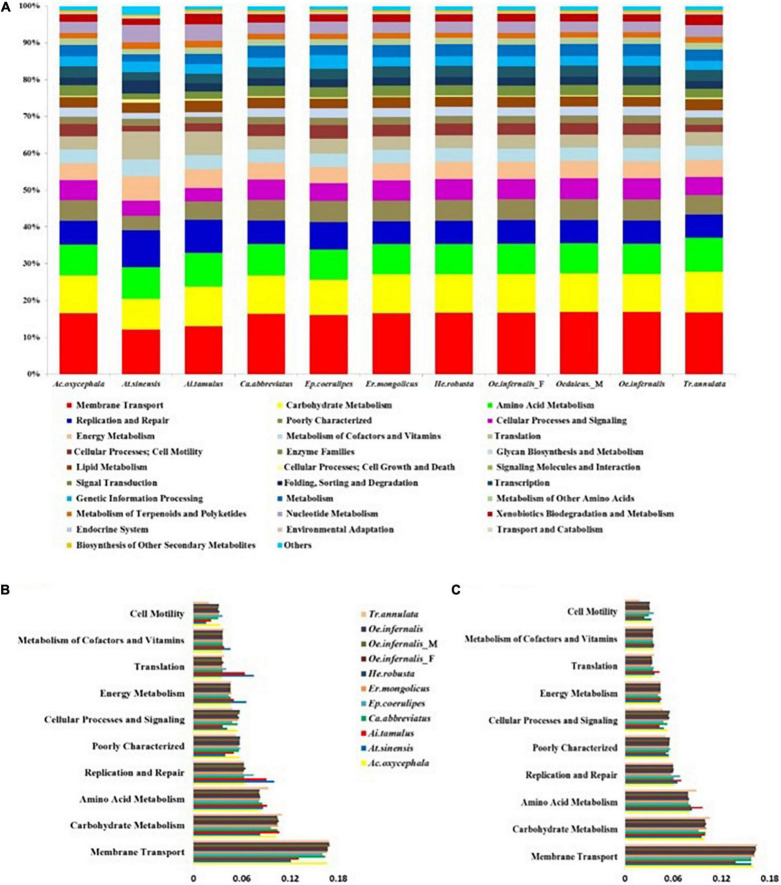
The predicted functions of gut bacteria in grasshoppers using PICRUSt. **(A)** Functional prediction of gut bacteria in all grasshoppers. **(B)** Histogram showing the 10 dominant KEGG pathways of the common 18 OTUs. **(C)** Histogram showing the 10 dominant KEGG pathways of gut bacteria in all grasshoppers.

Based on 16S rRNA sequence and KEGG database, PICRUSt was conducted to predict the metabolic pathways involved in the gut bacteria of grasshoppers. Using this method, 41 KEGG pathways were found involved by all gut bacteria in the nine grasshopper species (Figure 4A). The 10 dominant KEGG pathways found in all gut bacteria and the common 18 OTUs in nine species of grasshoppers were identical, the majority of which belonged to membrane transport, carbohydrate metabolism, amino acid metabolism, replication and repair, poor characterization, cellular processes and signal, energy metabolism, translation, metabolism of cofactors and vitamins, and cell motility (Figures 4B,C). In addition, *T. annulata* had a higher proportion of three KEGG pathways (membrane transport, carbohydrate metabolism, and amino acid metabolism) than other grasshoppers.

## Discussion

Grasshoppers, one of the most important primary consumers, have enormous ecological and economic value. Herbivorous grasshoppers rely on their gut bacteria to extract nutrients from indigestible plant tissue (Muratore et al., 2020a). However, when compared to the study of agricultural pest prevention and control, there are few studies on gut bacteria in grasshoppers. In the current study, we investigated the composition and function of the gut bacterial communities of nine grasshopper species in the Loess Plateau in northern Shaanxi, China. Firmicutes and Proteobacteria dominated the gut bacteria of nine grasshopper species. At the genus level, the detailed composition of these grasshoppers varied greatly. The highest proportion of the genera in Firmicutes was *Bacillus*, *Enterococcus*, and *Lactococcus*, respectively, and the highest proportion of the genera in Proteobacteria was *Enterobacter*, *Pantoea*, *Halomonas*, respectively. Several other herbivores have been found to have a similar composition of gut bacteria. For example, *Enterobacter*, *Bacillus*, and *Pantoea* are also found in the herbivorous *Hooded crane* (Zhao et al., 2017). The predominance of the gut bacteria of the herbivorous *Spodoptera* also includes *Enterococcus* and *Pantoea* (Chen et al., 2016). *Enterobacter* was also found in the silkworm and other herbivorous insects (Liang et al., 2015). *Enterobacter* has the ability to produce 1, 4-β-xylosidase, which is involved in the degradation of the plant cell wall (Suen et al., 2010). *Bacillus* can produce the endoglycanase enzyme required for the degradation of cellulose, which has the effect of degrading cellulose (Wakarchuk et al., 1994). *Lactococcus* can secrete expressive proteins (antigens or antibodies) that can interact directly with the substrate or the immune system of the intestinal mucosa (Hanniffy et al., 2007).

The functional contribution of the insect’s gut bacteria to its behavior and survival is indisputable (Dillon et al., 2000). Among grasshoppers, the highest proportion of the common 18 OTUs was *Bacillus*, *Enterobacter*, and *Enterococcus*. We used the PICRUSt to infer the function of all gut bacteria and the common 18 OTUs of nine grasshopper species based on 16S rRNA sequencing. It was found that the dominant functions of all gut bacteria were the same as the common 18 OTUs of all individuals, and these functions are membrane transport, carbohydrate metabolism, amino acid metabolism, etc., which are closely related to food transportation, digestion, and nutrient absorption. The results revealed that the common 18 OTUs of gut bacteria in nine grasshopper species are the basic components of gut bacteria in grasshoppers. However, due to the limitation of PICRUSt, the result of functional predictions should be cautious (Corby-Harris et al., 2014). For example, PICRUSt could only predict the OTUs that were matched with the available database and could not predict novel and unstudied OTUs (Langille et al., 2013). Many practical functions of gut bacteria of grasshopper are still worthy of being discovered and studied.

We found that although these grasshoppers have same ecological environments (such as climate, temperature, etc.); the composition of gut bacterial communities varies greatly across species. This indicates that the host species was an important factor for the gut bacteria community structure. This is consistent with previous results (Yang et al., 2016). The diversity of gut bacteria communities in *T. annulata* is greater than in the other eight species, as is the number of OUTs in *T. annulata*. *T. annulata* is a widely distributed insect in Asia, from east of Japan, west to Pakistan, and India (Bai et al., 2016; Guan and Xu, 2016). *T. annulate* has a more varied diet than other grasshopper species, so it may need more abundant gut bacteria to adapt the diversification of environment. A better understanding of these important host–microbiome interactions will provide insights into both host ecology and bacterial functions, potentially leading to better conservation of these species and their habitats (Muratore et al., 2020a).

## Conclusion

Here we demonstrated the composition and function of the gut bacterial communities of nine grasshopper species in the Loess Plateau in northern Shaanxi, China. The dominant bacteria genera of all the samples are *Enterobacter*, *Pantoea*, and *Halomonas*. The differences in the composition of gut bacterial communities of the nine grasshopper species may be affected by the host species and the dietary diversity. Despite the fact that our sample was not representative of age, sex, or physiological status, all of them are factors affecting the gut bacteria composition of insects. Our study provides a meaningful reference for the future study of gut bacteria in grasshoppers.

## Data Availability Statement

The datasets presented in this study can be found in online repositories. The names of the repository/repositories and accession number(s) can be found in the article/Supplementary Material.

## Author Contributions

LZ, W-QW, D-LG, and S-QX conceived the study and designed the experiments. LZ and D-LG performed the experiments, analyzed the data, and wrote the manuscript. D-LG, S-QX, and W-QW revised the manuscript. S-QX and D-LG are the co-corresponding authors. All authors read and approved the final manuscript.

## Conflict of Interest

The authors declare that the research was conducted in the absence of any commercial or financial relationships that could be construed as a potential conflict of interest.

## Publisher’s Note

All claims expressed in this article are solely those of the authors and do not necessarily represent those of their affiliated organizations, or those of the publisher, the editors and the reviewers. Any product that may be evaluated in this article, or claim that may be made by its manufacturer, is not guaranteed or endorsed by the publisher.

## Footnotes

1
http://greengenes.lbl.gov/Download/Sequence_Data

2
http://drive5.com/uparse/

3
http://rdp.cme.msu.edu/
