# Supplementary figures and images for: The Comparison of Gut Bacteria Communities and the Functions Among the Sympatric Grasshopper Species From the Loess Plateau
Source: Front Microbiol. 2022 Apr 5;13:806927. doi: 10.3389/fmicb.2022.806927 (PMC9037097; doi:10.3389/fmicb.2022.806927)

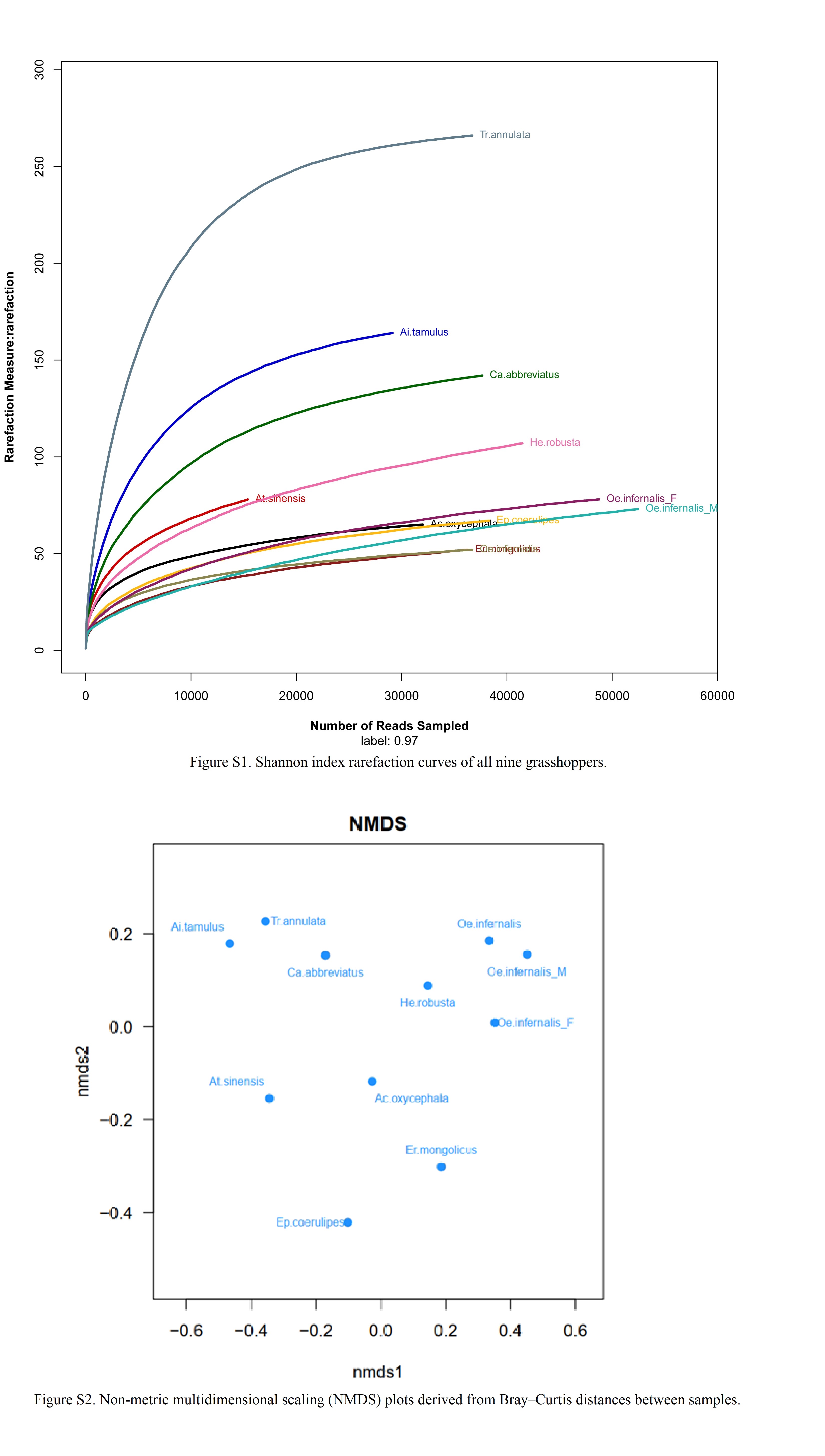

Supplement: Supplementary file 2 [file Image_1.JPEG]
